# Preparation and Evaluation of Rifampicin and Co-trimoxazole-loaded Nanocarrier against *Brucella melitensis* Infection

**DOI:** 10.22034/ibj.22.4.275

**Published:** 2018-07

**Authors:** Narges Bodaghabadi, Samira Hajigholami, Ziba Vaise Malekshahi, Maliheh Entezari, Farhood Najafi, Hadi Shirzad, Majid Sadeghizadeh

**Affiliations:** 1 Molecular Genetics Department, Faculty of Biological Science, Tarbiat Modares University, Tehran, Iran; 2 Department of Medical Biotechnology, School of Advanced Technologies in Medicine, Tehran University of Medical Sciences, Tehran, Iran; 3 Department of Biology, Tehran Medical Sciences Branch, Islamic Azad University, Tehran, Iran; 4 Department of Resin and Additives, Institute for Color Science and Technology, Tehran, Iran; 5 Medical Genetic Department, Faculty of Medical Sciences, Tarbiat Modares University, Tehran, Iran

**Keywords:** Brucellosis, Cytotoxicity, Nanoparticles, Rifampicin

## Abstract

**Background:**

Brucellosis or Malta fever is a contagious infection common between human and domestic animals. Many antibiotics are used for brucellosis treatment, but they are not efficient and put heavy burden on society. Co-trimoxazole and rifampicin are two candidates for brucellosis treatment. In this study, we aimed to enhance the efficacy of these antibiotics using designed nanoparticles.

**Methods:**

Different concentrations of co-trimoxazole and rifampicin were used for loading onto a nanostructure of synthesized monomethoxy poly(ethylene glycol)-oleate (mPEG-OA). The solubility, cytotoxicity, and efficacy of these nano-packed antibiotics on *Brucella*-infected murine phagocytic cells were examined, as compared with free antibiotics. Then the release nanoparticles was increased approximately 3.5 and 1.5fold, respectively, which is considerable in comparison with free insoluble ones.

**Results:**

Despite acceptable loading percentage, the application of co-trimoxazole-loaded nanoparticle on *Brucella*-infected J774A.1 murine macrophage-like cells did not lead to reduction in the number of bacteria; however, the efficacy of rifampicin on *Brucella*-infected murine phagocytic cells enhanced.

**Conclusion:**

In the current study, the efficacy of rifampicin on reducing the number of *Brucella melitensis* increased by the novel synthesized nanostructure. In contrast, since co-trimoxazole efficacy did not enhance by loading onto nanoparticles, the co-trimoxazole inefficiency is most likely not due to its low penetration or insolubility, and probably there are other factors that remain to be clarified in the future investigations.

## INTRODUCTION

For many years, infectious diseases have been one of the main causes of death worldwide[1]. Brucellosis, as an infectious disease, is caused by Gram-negative, non-motile, and facultative intracellular bacteria[2]. Its annual infection rate is approximately 500,000 people in the world[3]. There are six classical species of *Brucella*, of which *B. melitensis* is the most important because it causes severe diseases in human[4].

Many antibiotics have been developed to combat such bacterial infections, however, some crucial obstacles such as low water solubility, rapid clearance rate, insufficient penetrance across the cell membrane, cytotoxicity, and emergence of antibiotic resistancy can weaken their efficacy[5].

Rifampicin, as a drug for brucellosis, has low solubility in water and causes precipitation and rapid clearance from the body. Increasing hepatotoxicity, inefficiency, and treatment costs are direct consequences of consuming multiple doses of rifampicin[6]. Co-trimoxazole, a low-cost drug, also has not shown favorable results.

Using nanoparticles, as a promising solution to the problem of antibiotic inefficiency, has received the attention of many researchers. By the use of nanoparticles, drug penetration across the cell membrane is accelerated. There are other positive features in these nanostructures that may obviate many obstacles in medical treatment when used as a nano-vehicle for antimicrobial agents. For instance, by using nanoparticle properties, targeted therapy is now achievable[7,8].

Previous studies by our group showed that dendrosome, a diblock nanostructure made from oleic acid (OA) and polyethylene glycol (PEG, 400 or 2000 Dalton), has indirect anticancer effects on cells by inducing apoptosis pathways and reducing tumor size in mice[9,10]. It has also been shown that this nanoparticle has DNA transfection ability[11,12]. Based on the critical micelle concentration measurement, loading and encapsulation efficiencies, and other cellular experiments, this novel nanoparticle has been considered as an appropriate drug delivery system[1].

In this study, we exploited monomethoxy poly(ethylene glycol)-oleate (mPEG2000-OA) as a proper biocompatible, low-cost and well-defined physical compound for co-trimoxazole and rifampicin encapsulation, namely polymeric nanoparticle-co-trimoxazole (PNCT) and polymeric nanoparticle-rifampicin (PNR). Moreover, MTT assay was performed for evaluation of PNCT and PNR cytotoxicity, and the efficacy of antibiotic-loaded nanoparticles were tested against intracellular *Brucella* bacteria.

## MATERIALS AND METHODS

### Bacterial strain, cell line, and antibiotics

*B. melitensis* 16 M was grown in *Brucella* agar at 37°C with 5% CO_2_. For cell culture studies, J774A.1 murine macrophage-like cells (ATCC) were grown in DMEM with 10% heat-inactivated fetal bovine serum and 1% penicillin-streptomycin (Cellgro, USA) in a humidified atmosphere of 5% CO_2_ at 37°C. Rifampicin and co-trimoxazole were purchased from Hakim Pharmaceutical Co. (Tehran, Iran) and Tehran Chemie Pharmaceutical Co. (Tehran, Iran), respectively.

### Preparation of antibiotic-loaded nanoparticle

Antibiotics were loaded into nanoparticle by co-solvent evaporation method. In brief, antibiotics were dissolved in 3 ml solvent with low evaporation point (sulphamethoxazole in acetone, trimethoprim in methanol, and rifampicin in methanol), and nanoparticle was dissolved in 1 ml PBS; then solvents were mixed. By utilizing Heidolph Rotary Evaporator, only the solvents of rifampicin and co-trimoxazole were evaporated, and the nanocarrier was reformed; in which rifampicin and co-trimoxazole, as hydrophobic antibiotics, were confined in hydrophobic nanoparticle interior shell. In the presence of methanol or acetone, nanocarrier lost its integrity. PNCT and PNR were filtered to prevent the free aggregated antibiotics contaminates. Afterwards, the PNCT and PNR powder forms were obtained by freeze drier (Snijders Scientific, Tilburg, Holland).

To obtain loading efficacy, 10 mg powder was weighed and solved in methanol, and the amount of loaded antibiotics was calculated from concentration values obtained by the calibration curve of the rifampicin and co-trimoxazole at 475/507 nm and 271 nm (T80+ UV/VIS Spectrometer; PG Instruments Ltd., UK), respectively. Another control was also included in which nanocarrier was absent from the procedure, which was further subtracted from the equation of drug loading and encapsulation efficacy. To determine optimum concentration of antibiotics in loading, different concentrations (2, 4, 6, 8, 12, 16, 20, and 25 mg/ml) of antibiotics were loaded onto 100 mgml^-1^ nanocarrier. By obtaining the amount of the drug entrapped in the nanoparticle, the best concentration of antibiotics for loading was determined. The drug loading and entrapment efficiency were calculated by the following equations:


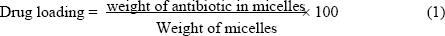



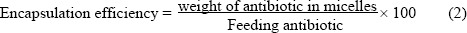


By considering that hydrophobic drug would be entrapped in biocompatible nanocarrier, augmentation in solubility and the absorbance of co-trimoxazole- and rifampicin-loaded nanocarrier was expected. For the absorption spectra (300-700 nm) of free and loaded co-trimoxazole rifampicin at the concentration of 1 mg/ml, they were measured by Thermo Scientific™ NanoDrop (USA).

#### In vitro cytotoxicity

MTT assay was performed to determine the cytotoxicity of the empty nanocarrier, free antibiotics, and antibiotic-loaded nanocarrier. Briefly, J774A.1 ATCC (10^4^/well) was seeded onto 96-well tissue culture plates (Corning Inc., USA) containing DMEM, 10% heat inactivated fetal bovine serum (FBS), and 1% penicillin-streptomycin (Cellgro, USA). After 24-hour incubation, free drugs, empty nanocarrier, and antibiotic-loaded nanocarrier were seprately added to cells in a serial concentration to each well and incubated for 24 h. Untreated cells were considered as negative controls. Samples were tested in triplicate. After 24-hour incubation, 20 µL MTT [3-(4,5-Dimethyl thiazal-2-yl)-2,5-Diphenyltetrazolium Bromide] solution (Promega, USA) was added to each well. The plate was further incubated in the dark 5% in CO_2_ atmosphere at 37°C for 4 h, and the medium of each well was replaced with 200 µl DMSO. Twenty minutes after DMSO treatment, the absorbance at 570 nm was measured using a 96-well ELISA plate reader. Finally, the absorbance ratio was calculated for the viability of samples.

#### *In vitro* infection assay

**Fig. 1:**
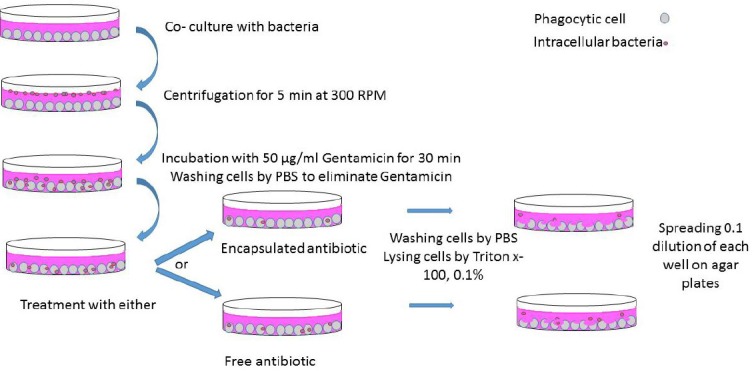
Schematic description of infection and antibiotic treatment.

As shown in Figure 1, macrophages were seeded at a density of 10^5^ cells/well onto a 24-well cell culture plate (Corning Inc., USA) 24 hours prior to infection. Then the cells were infected with *B. melitensis*16 M (stock cultures) at the ratio of 1:100. Subsequently, the medium was removed, and the cells were washed three times by a fresh medium containing 10% FBS and 50 µg/ml gentamicin. After washig steps, an appropriate amount of medium was added, and the cells were incubated at 37°C for another 24 hours. Following the incubation, cells were washed three times with PBS to eliminate any remaining gentamicin. Serial concentrations below cytotoxic threshold of nanocarrier, free or encapsulated co-trimoxazole, and rifampicin were added to each well along with a medium containing 10% FBS. After 24-hour incubation, the medium was removed, and the cells were washed twice with PBS, lysed by 0.1% Triton-X100, and 10-fold serial dilutions of lysate were cultured on the *Brucella* agar plate. After 72-hour incubation at 37ºC and 5% CO_2_, the bacterial count was calcuated. Using the latter assay, the efficacy of the antibiotic-loaded nanocarrier compared with free antibiotic against intracellular *Brucella* was tested.

#### *In vitro* drug release

*In vitro* drug release study of effective drug-loaded nanocarrier was measured by a dialysis bag (MWCO 12–14 kDa, pore size of 2.4 nm). Two dialysis bags were repleted by the same concentration of rifampicin-loaded nanocarrier and free rifampicin and immersed in 10 mL PBS at two different pH values (5.2 and 7.4), which represent endosomal pH of macrophages and physiological pH, respectively. Ascorbic acid (0.2% w/v) as an antioxidant agent was added to the solution to prevent rifampicin degradation[13]. The whole system was stirred at 100 rpm and kept at 37°C, similar to human blood circulation speed and temperature. At determined times, 1 ml of samples was withdrawn and replaced with a fresh medium. Absorbance of samples was measured at 475 nm using T80+ UV/VIS Spectrometer (PG Instruments Ltd., UK).

### Statistical analysis

All data were analyzed using Microsoft Excel, and One-way ANOVA test was used to determine *P* values. A *P* value <0.05 was considered statistically significant.

## RESULTS

### Preparation of antibiotic-loaded nanoparticle

**Fig. 2:**
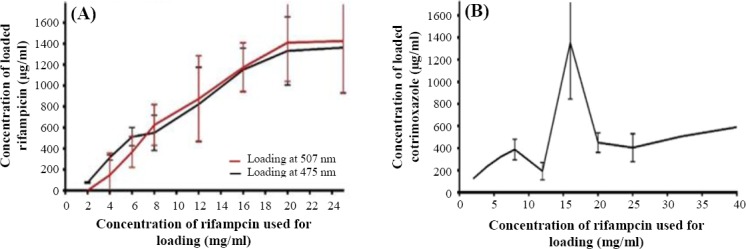
Determining optimum concentration of antibiotics for efficient loading. Different concentrations of antibiotics with 100 mg/ml nanocarrier were used for loading, and then their antibiotics payloads were measured. As shown in the Figure, 16 mg rifampicin (A) and co-trimoxazole (B) with 100 mg nanocarrier have the best loading efficiency.


To optimize the best concentration of feeding antibiotics for highest loading efficiency, different concentrations of co-trimoxazole and rifampicin (2, 8, 12, 16, 20, 25, and 40 mg/ml) were loaded onto 100 mg/ml nanoparticle, and then the antibiotic payloads were measured. The optimum concentration of antibiotics for loading was 16 mg/ml with 100 mg/ml nanoparticle. This concentration of antibiotics not only has the highest drug loading efficacy but also eliminates the cost of using extra antibiotics. Loading, as described above, was performed, and the loading efficiency of approximately 30% was gained, which is considerable (Fig. 2). As the loaded antibiotic is freeze-dried, this stable form of drug can facilitate its long-time storage. Drug loading and encapsulation efficiency were calculated based on the following formulas:

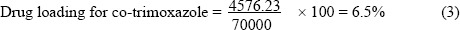



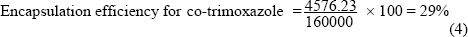


Some proportions of the feeding antibiotics were on the nanoparticle surface that were subtracted in calculating encapsulation efficiency and drug loading. These proportions of antibiotics were determined using a control sample in which the nanoparticle was absent in the loading procedure.


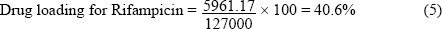



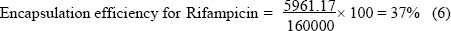


### Loading confirmation

**Fig. 3:**
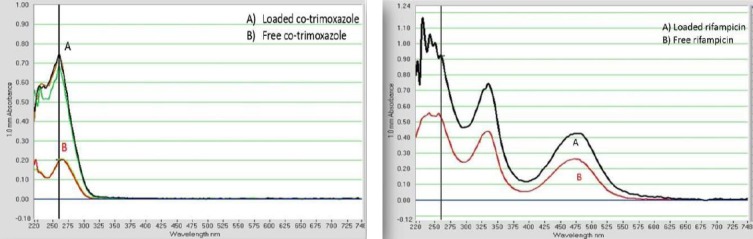
Absorption spectrum (300-700 nm) of (A) loaded antibiotics (B) free antibiotics. Antibiotic-loaded nanocarrier has approximately twofold more solubility than free antibiotic in water measured by Thermo Scientific™ NanoDrop.

In this research, solubility enhancement of co-trimoxazole and rifampicin was expected as a result of loading. To determine this, the absorbance of both free antibiotics and antibiotic-loaded nanoparticle at the same concentration was measured at 200-700 nm by Thermo Scientific™ NanoDrop. Our evaluations showed approximately twofold more solubility than free antibiotic in water (Fig. 3). Soluble antibiotic-loaded nanocarrier had a transparent solution, while free antibiotics with the same concentration were precipitated.

#### *In vitro* cytotoxicity

**Fig. 4:**
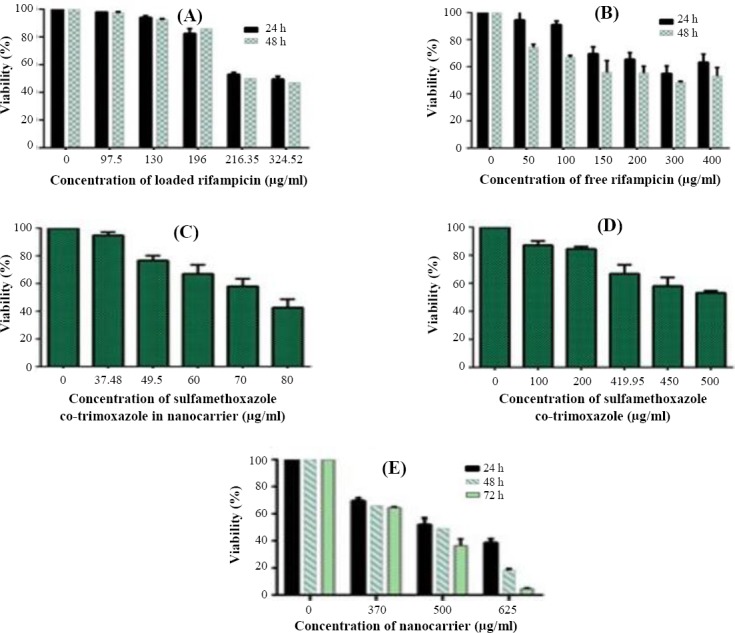
Effect of antibiotic-loaded nanocarrier (A [rifampicin] and C [co-trimoxazole]) and free antibiotics (B [rifampicin] and D [co-trimoxazole]) nanocarrier (E) on the viability of J774A.1 cells. Incubated cells with media were used as positive controls. All treatments were tested in three independent experiments.

MTT assay was performed to obtain cytotoxic concentration. Compared with untreated controls, no significant toxicity was observed for applied concentrations of neither free antibiotics nor antibiotic-loaded nanocarrier on J774A.1 macrophages (Fig. 4). In other words, concentrations used in the following experiments were much lower than *in vitro* cytotoxic concentration for macrophages.

#### *In vitro* efficacy against *B. melitensis*

**Fig. 5:**
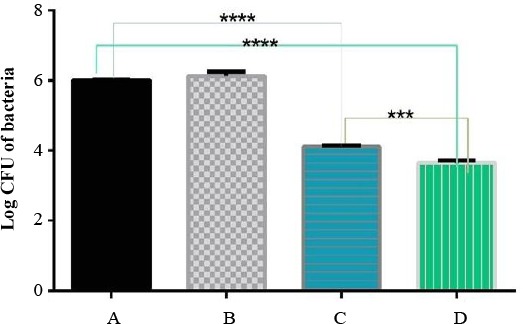
Efficacy of different threats against B. melitensis. CFU of B. melitensis as treated by (A) untreated samples, (B) nanocarrier, (C)free rifampicin, and (D) loaded rifampicin. ^***^P=0.0006; ^****^P<0.0001

*Brucella*-infected J774A.1 murine macrophage-like cells were treated by a serial concentration, less than cytotoxic threshold of free antibiotics or antibiotic-loaded nanoparticles. After 24 hours, cells were lysed, and the intracellular number of *Brucella* was counted. Data were presented as the mean±SEM of four independent experiments. As the advantages of encapsulation technology are numerous including sustained drug release and evading the immune system, efficient reduction in the number of bacteria was expected. Nanocarrier-treated samples did not show any reduction in the number of bacteria. This means that in samples treated with antibiotic-loaded nanocarrier any observed reduction in the number of bacteria is due to the increased efficacy of antibiotics, but not the nanocarrier. Here, the efficiency in reduction of intracellular bacteria treated with antibiotic-loaded antibiotic samples. Rifampicin-loaded nanocarrier and free rifampicin revealed significant reduction of bacterial number in comparison with negative controls, and loaded rifampicin showed greater efficacy than free one (Fig. 5). However, reduction in the number of bacteria neither with free co-trimoxazole nor with co-trimoxazole-loaded nanoparticle was not observed at high concentration.

### *In vitro* drug release

**Fig. 6:**
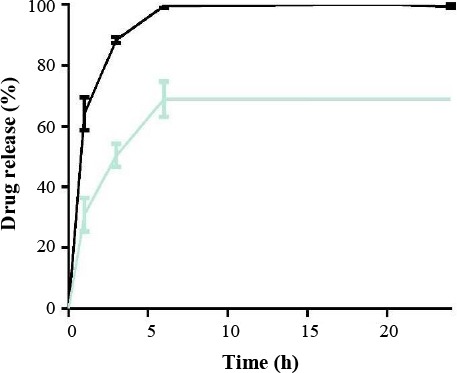
Drug release at pH 7.4. Approximately, 60% of the drug was immediately released after six hours, in both free rifampicin and rifampicin-loaded nanocarrier.

Release studies, in two different pH solvents, revealed that rifampicin release from nanoparticles in an effective complex would be pH-independent. Approximately 60% of the drug was immediately released after six hours, in both free rifampicin and rifampicin-loaded nanocarrier (Fig. 6).

## DISCUSSION

In intracellular infections, bacteria evade killing mechanisms of immune system and antimicrobial drugs because they are protected by membrane barrier of infected cells. Few studies have been conducted on functional antibiotics in phagocytic cells, but the observed reduction of bacterial numbers in antibiotic-treated phagocytic cells strongly confirms the therapeutic efficacy of these antimicrobial compounds. Antibiotic-loaded nanocarriers could reach the intracellular niche of bacteria, where they are able to release their content near their targets. In this sense, a meaningful reduction in the number of intracellular bacteria that were treated with antibiotic-loaded nanocarriers in comparison with free antibiotic samples might occur as an expectable result.

The use of nanoparticles, as an antimicrobial drug vehicle, will overcome this problem to some extents. Although up to now, no antibiotic on a nano basis has been declared to cause complete clearance of intracellular bacteria, loading antibiotics onto nanocarriers can lead to increased solubility, reduced cytotoxicity, and finally improved treatment of patients, even though complete clearance of bacterial cells does not occur; This stimulates researchers to apply studies on nanoparticles[7,8].

Despite the fact that nanodrugs studies against intracellular bacteria are still in their beginning phases, numerous nanocarriers with the capacity of loading antimicrobial drugs have been synthesized[14]. The inefficiency of antibiotic regimen for human brucellosis partially depends on bacterial resistance. Over time, bacteria have learned to evade killing mechanisms of not only their host immune system but also antimicrobial agents[15]. In many cases, stronger antibiotics have been synthesized to overcome the problem of drug-resistant bacteria. However, these approaches have faced limited success and even in some cases greater resistance[16,17]. As a result, synthesizing stronger antibiotics for overcoming resistant bacteria might fail because of innovative mechanisms by which bacteria escape the therapeutic approach. The inefficiency of antibiotics also leads to high-consuming dose and therefore, cytotoxic effects.

Rifampicin is a widely used antibiotic both for brucellosis and for a wide range of diseases including tuberculosis[18]. Decreased number of bacteria treated with free rifampicin is due to eradicating some extracellular bacteria and those that are transferred to other cells via life cycles[19].

In this study, the capability of mPEG-OA, as an antibiotic carrier, was determined for the first time in order to increase the efficacy of antibiotics in treatment of an intracellular bacterium. Applicable results were achieved for rifampicin as an antibiotic for the intracellular bacteria *Brucella*, although further studies focusing on treating other intracellular infections by different antibiotic-loaded nanocarriers or different drugs should be conducetd to confirm this finding. In the case of co-trimoxazole, we aimed to enhance co-trimoxazole efficacy by using nanoparticle for the first time. We did not obtain any reduction in the number of bacteria even when it was loaded onto nanoparticle. Therefore, we conclude that despite current prescription of co-trimoxazole for brucellosis treatment, it is not a proper antibiotic even if its translocation across the cell membrane is accelerated[9], the release rate is controlled, and the solubility is increased via nanoparticle.

The results indicated that a proportion of the drug may be on the surface of nanoparticles that is exactly similar to the amount of antibiotic obtained by control (data not shown). In this sense, the latter amount was subtracted from encapsulation efficacy and drug loading. It is predictable that the remaining encapsulated drug can be released following entry into the cell. Therefore, using a nanocarrier, as a vehicle of rifampicin, provides an advantage because drug release can be controlled, and the need of prolonged treatment of Philippon and coworkers[20], 300 mg/kg/day orally administrated co-trimoxazole for 19 days did not show any improvement in patients’ health. Other clinical results and *in vivo* experiments with this antibiotic were also unsuccessful[15,21]. Co-trimoxazole, as a low-cost antibiotic, is widely used for brucellosis treatment, especially in developing countries, while high resistance against co-trimoxazole has been reported (62%)[22,23].

*Brucella* is an intracellular bacterium; therefore, we cannot conclude the *in vivo* sensitivity of *Brucella* to antibiotics from *in vitro* results. Overall, in this study,

*in vivo* experiments of rifampicin- and co-trimoxazole-loaded nanoparticle treatment are mandatory. As these complexes may release, their contents after entering the cell, *in vivo* studies will give comprehensive knowledge about the efficiency of antibiotics, and obtaining more positive results is feasible.

A new approach for dealing with the emergence of resistance bacteria against antibiotics is the use of nanoparticles as a carrier for the antibiotic. The results of the current study demonstrate that it is worth working on this promising approach. With the development of numerous nanocarriers with the capacity of loading antimicrobial drugs, many more combinations of antibiotics and nanocarriers can be studied.
